# “It’s just one piece of the puzzle…”: practitioners’ perspectives on the use of sterilisation in the management of free-roaming dog populations

**DOI:** 10.3389/fvets.2026.1898448

**Published:** 2026-08-18

**Authors:** Abi Collinson, Marnie L. Brennan, Rachel S. Dean, Jenny Stavisky

**Affiliations:** 1 School of Veterinary Medicine and Science, University of Nottingham, Nottingham, United Kingdom; 2 VetPartners, York, United Kingdom

**Keywords:** community engagement, dog population management, free-roaming dogs, One Health, surgical sterilisation

## Abstract

Surgical sterilisation is a common approach to managing free-roaming dog (FRD) populations, used predominantly with the aim of reducing or stabilising the population. However, monitoring and evaluation of the impacts of such programmes is limited and there can be a tendency to equate numbers of dogs sterilised with success. The aim of this study was to examine practitioners perspectives on the role of sterilisation in managing FRD populations, including insights into its perceived benefits and limitations, and the wider facilitators and challenges that shape these efforts. Purposive convenience sampling was used to recruit 15 programme staff from 12 organisations working in dog population management (DPM) globally, although Africa and the Middle East were not represented. Semi-structured interviews were undertaken and explored experiences of managing FRD populations through sterilisation. Reflexive thematic analysis generated four themes: (1) ‘Seeing only part of the problem’ describes complexities in relation to FRD populations that are frequently overlooked when planning and designing programmes; (2) ‘Not a silver bullet’ illustrates participant experiences of outcomes not materialising as expected after the introduction of a sterilisation programme; (3) ‘Looking beyond the dogs’ illustrates an increasing awareness of the importance of human factors in dog population management, rather than interventions that are solely focused on dogs; and (4) ‘Sterilisation within a wider strategy’ describes key implementation facilitators perceived as important for programme success. Reframing DPM as a ‘wicked problem’ that does not have a simple solution could help in the development and management of sustainable interventions. This would avoid oversimplification of the problem, and therefore the solution, and instead address these complexities to enable more impactful outcomes.

## 1 Introduction

The global domestic dog (*Canis familiaris*) population is estimated at between approximately 700 million–1 billion (1, 2) of which at least 75% are thought to be free-roaming (3). In addition to concerns regarding impaired welfare of free-roaming dogs (FRDs) such as high mortality (4, 5) and morbidity, they have also been associated with negative consequences for human health and wellbeing (6) and wildlife populations (7–9).

The aims of a DPM programme may include reducing or stabilising population size, improving dog health and welfare, reducing risks to public health and reducing conflict with livestock and wildlife (3, 10). There are various approaches that are used (11), with culling historically being the most common method for reducing numbers (11, 12). However, this is now regarded to be ineffective in the long term (3), as populations are capable of quickly rebounding to pre-culling levels (13, 14), and tend to be skewed towards younger dogs due to new births (14, 15). Animal welfare organisations have promoted sterilisation as a preferred option to culling, but acceptance of this change has been based on the assumption that controlling reproduction is the key factor governing population size.

There is a relatively small amount of published data on the long-term impacts of sterilisation programmes [reviewed by (11)]. The effects on population size and density are not consistent, with variable consequences reported including population decreases (16–18) and increases (19). However, results are complicated by variations in context and implementation such as dog population dynamics, sterilisation coverage and length of time of both programme implementation and follow-up population monitoring. A study from Brazil using a control area found no differences in abundance, survival and recruitment between sterilisation and non-sterilisation areas (20) indicating that sterilisation did not affect the population dynamics. However, this was only conducted and assessed over a 14 month period. The only randomised controlled trial of sterilisation found no significant differences in adult FRD counts between intervention and control sites during a 2-year follow-up period (21) though, there was a reduction in puppies and lactating females suggesting a lower population turnover and likely benefits for FRD welfare such as a reduction in puppy mortality.

Similarly, there is some evidence for positive effects of sterilisation interventions on canine welfare and public health (11, 22), such as reduced dog bites, dog and human rabies cases and improved dog health and welfare indicators and public perception or behaviour towards FRDs. Again though, this understanding is derived from a relatively small number of studies, which tend to focus on single outcomes in specific areas. This fails to capture complexities concerning context, implementation and the underlying mechanisms which lead to any reported benefits. Equally, research conducted which suggests limited benefits may fail to capture elements of programme implementation that lead to success. As a result, the production of useful knowledge and evidence that can be used in practice is limited. There is still sometimes a tendency within the wider field of DPM to equate numbers of dogs sterilised (outputs) with success (outcomes) (22), and an expectation that the more dogs that are sterilised, the greater the effects on the dog population and other associated benefits.

In order to evaluate complex interventions such as sterilisation for FRD management, a theoretical understanding of how the intervention causes change is needed (23). Key questions to be addressed are whether and how initiatives are effective in practice, as well as an understanding of the whole range of effects that may be produced and how they may vary in different settings (23). These relationships between context, implementation and mechanisms are important to identify and are multidirectional as implementation is affected by the existing context, but an intervention may change aspects of the context in which it is delivered (24).

To gain an understanding into how and why these programmes work in practice, qualitative methods are needed. The benefits of this approach have been demonstrated in evidence based medicine (25). Findings can help bridge the gap between research evidence and practice by providing insights into how interventions work in real world contexts and in understanding barriers and facilitators to successful outcomes. This expert and experiential knowledge can help inform and guide decision making (26). The use and value of these methods have often been overlooked in veterinary research in general (27), and certainly in DPM research to date.

This study aimed to gather stakeholder opinions and experiences from the perspective of those working in the field regarding the motivations, implementation and outcomes of using sterilisation within DPM programmes.

## 2 Materials and methods

### 2.1 Methodological approach

The study took a pragmatic approach to evaluate ‘what works’ in practice and develop that knowledge by mapping connections between experiences, knowledge and action (28). This approach was also aligned with the intention to contribute useful and actionable knowledge based on participants’ experiences that could help to develop practical solutions and inform future practice (29).

### 2.2 Participants and recruitment

A purposive convenience sample of DPM practitioners (including programme founders, managers, veterinarians) whose programmes involved sterilisation of FRDs, and who were involved in decision-making regarding these programmes, was used for this study. Purposive sampling allows for the identification and selection of information-rich cases, defined as “those from which one can learn a great deal about issues of central importance to the purpose of the inquiry” (30). Participants were selected to represent the use of sterilisation in diverse contexts, in terms of geographical setting, FRD population characteristics, perceived problems with FRDs and strategies used. Practitioners were identified from the authors professional networks, as well as those who had taken part in a previous study (31) (*n* = 152), and had expressed an interest in being interviewed for future research (*n* = 71). Participants were contacted through email. Additional practitioners were recruited through snowball sampling if a recommendation was made. Participation was voluntary and no incentives were offered. Table 1 describes the details of organisations represented by interview participants. Their roles were predominantly in programme management, but 7 were also veterinarians or veterinary technicians. The organisations were based in Australia, Canada, USA, India (*n* = 2), Nepal, Afghanistan, Thailand, Bali, Panama, Chile and Romania. Organisations varied as to whether they worked from one location at a static clinic and/or shelter or if they worked at several different locations via a mobile clinic or field clinics, or a combination of both. The time the programme had been operating ranged from 3 to 27 years with a median of 16 years.

**Table 1:** Details of organisations represented by interview participants.

Region	Type of intervention	Setting	Years since intervention started
Oceania	Multiple locations—field clinics	Rural	18
Asia	Static clinic and field clinics	Urban and rural	16
Americas	Multiple locations—field clinics	Rural	8
Americas	Static clinic	Urban and rural	12
Asia	Static clinic and field clinics	Urban and rural	11
Asia	Static clinic	Urban	27
Asia	Static clinic	Urban	3
Americas	Multiple locations—field clinics	Rural	15
Asia	2 static clinics and mobile clinics	Urban and rural	18
Americas	3 static clinics and field clinics	Urban and rural	20
Europe	Static clinic and field clinics	Urban and rural	19
Asia	Static clinic	Urban	16

Sample size was guided by the concept of information power (32), which suggests that when the research question is highly relevant to participants’ experiences, fewer participants are needed. In this study, participants had highly relevant experience, the interviews were in depth and analysis generated meaningful insight, therefore the dataset was deemed to hold sufficient information power.

### 2.3 Data collection

Data was collected using semi-structured interviews. Prior to the interviews being conducted, participants were provided with a study information sheet and were required to read and sign the study consent form. Interviews were conducted by AC via video or audio call using either Microsoft Teams or Zoom depending on participant preference. Participants were either at home or at their place of work during the interview. Interviews followed an interview guide consisting of open-ended questions (Supplementary material S1) covering reasons for using sterilisation, perceptions of what had, or had not, happened as a result of the programme and other factors considered important for success. A pilot interview was conducted with an employee of one of the organisations for which multiple interviewees were available, after which minor changes were made to the interview guide. Demographic information concerning the participant’s role and basic information regarding the organisation they worked for, such as length of time that it had been operating for, location(s) and sterilisation strategies employed was also collected.

Twelve interviews were conducted between July 2020 and May 2021, capturing the views of 15 programme staff from 12 organisations. One interview took place in dyadic (two-person) and one in triadic (three-person) format. The choice to include more than one individual in some of the interviews was made if it was considered by the initial contact at the organisation during the recruitment process that there was another person or additional people who it was felt could offer additional insight into organisational activities and processes. For example, the interview with 2 participants included both the founder/director of the organisation and the co-ordinator of the catch-neuter-vaccinate-release programme. This approach is consistent with the literature on joint and dyadic interviewing, which recognises that a strength of interviewing participants together is that it can maintain the depth and rich detail of individual interviews whilst providing the interaction present in focus groups (33). Interview length ranged from 41 min to 97 min.

### 2.4 Data analysis and reporting

Audio files were either auto-transcribed or transcribed verbatim by AC and then checked for accuracy against the original recording. Transcripts were not returned to participants. Notes were made on each interview to record key ideas or new insights collected in that interview. All potentially identifying information about the participant and their organisation was removed to protect participants’ anonymity.

Thematic analysis was carried out inductively by AC following the six-step plan described by (34) and managed using NVivo (NVivo qualitative data analysis Software; QSR International Pty Ltd. Version 12, 2018). A selection of three transcripts was inductively coded by JS to sense-check ideas and explore interpretations of the data. Codes were inductively grouped according to their content to form subthemes. Subthemes were repeatedly reviewed and refined during the analysis and writing-up process. The subthemes were then mapped onto a single large sheet of paper and manually organised into themes containing related subthemes.

This study is reported where appropriate according to the Consolidated Criteria for Reporting Qualitative Research (COREQ) guidelines (35).

### 2.5 Reflexive statement

The interviewer and primary author (AC) is a white, female vet who has spent substantial periods of time working for organisations in low- and middle-income countries which focus on sterilisation of FRDs. My background in this field could have affected the questions asked to reflect personal experiences, and also shaped my interactions with the interviewees. JS and MLB were also involved in the design of the interview guide, and a selection of interview transcripts were coded by JS. Ongoing meetings and discussions about the data and analysis took place throughout the research process. Differences in interpretation were discussed collaboratively to refine code and theme boundaries and enhance reflexivity and interpretative depth, rather than to achieve consensus between coders (36).

## 3 Results

Four themes were generated from the analysis. (1) ‘Seeing only part of the problem’; (2) ‘Not a silver bullet’; (3) ‘Looking beyond the dogs’, and (4) ‘Sterilisation within a wider strategy’. These are summarised in Table 2.

**Table 2:** Summary of the themes generated from the analysis of interviews with DPM practitioners describing their experiences of conducting sterilisation programmes.

Theme	Subthemes	Summary
Seeing only part of the problem	Action over insight Differing perspectives	FRD populations are not just a result of uncontrolled reproduction and the problem is not just that there are too many dogs
Not a silver bullet	The sterilisation treadmill Change beyond numbers	Sterilisation does not always lead to the expected results
Looking beyond the dogs	A means, not the end From co-existence to care	There is an increasing awareness of human behaviour change in achieving long term sustainable changes for FRDs
Sterilisation within a wider strategy	Co-creating change Developing veterinary capacity	The way in which a sterilisation programme is implemented is important

### 3.1 Seeing only part of the problem

This theme centres on the importance of recognising that managing FRD populations is a complex problem and describes some of the complexities that participants identified as often being overlooked during the initial conception and planning of a DPM programme.

#### 3.1.1 Action over insight

Participants felt that sterilisation programmes often began without a comprehensive understanding of the multi-causal nature of the problem. There was a tendency to focus instead on implementation and an emphasis on numbers of dogs sterilised as a marker for success. A typical experience has been that *“… [at the start] the big focus was just to spay and neuter everything.” (DPM3)*. There had also often been a feeling of needing to ‘help’ as many dogs as possible. This was usually a response to concerns regarding poor animal welfare and/or a need to provide a tangible alternative to the use of inhumane methods of DPM, e.g., culling. One participant described the situation in the city before their programme was established *“…at that time the municipality controlled stray dogs and [the policy] was to tie them up and leave them out in the desert.” (DPM12).*

Quantification of dog population size and demography prior to commencement of neutering was rare. This led to a situation where organisations had been trying to*“…fix[ing] the problem without first establishing that baseline of what the problem looked like” (DPM1)*. In addition, characterising the FRD population and understanding what sources were contributing to the population and what was maintaining it was seen as important step by some of the participants. This was considered key for being able to make informed decisions, e.g., different subgroups in the dog population may require different management approaches.

*“…a lot of people moved from the countryside to the town… and they let free their dogs and so started a problem with the stray(s)… And then we have another big problem that are the ones that are left free by people…owned dogs but they are left free to do whatever they want. So we have this mix of populations… and they are two problems” (DPM11).*

#### 3.1.2 Differing perspectives

Furthermore, there could be different perspectives of what the ‘problem’ was and therefore what ‘solving’ it would look like. This was described by one participant in relation to the long term goals of their programme.

*“This fact that there is no consensus on what you should do I think is a major hurdle. Whether you want them, whether you tolerate them, or whether you hate them. Because we can control the numbers, but where are we supposed to be going? Are we supposed to control numbers forever? Or are we heading for zero street dogs…” (DPM6).*

In addition, there were associated problems linked to FRDs such as human-dog conflict and risk of disease which may have their own or additional causes, other than simply being a result of a large dog population. For example, the issue might not necessarily be that there are too many dogs but could be either to do with a lack of access to resources or animal health services to care for them, described by one participant as *“the feeling of there being too many dogs… it’s not that they are not wanted… I feel like it’s more that they, the community members, just know they do not have the resources to take care of all of them…” (DPM8),* or could be a reflection of people’s perceptions of the perceived risk from particular dogs: *“We often get told there are too many dogs. I think a big contributor to that though is that they are free-roaming. So when you ask people how many dogs do you think there are in your community, the estimates that you get are incredibly exaggerated…because dogs are so visible in that community.” (DPM1).*

Misconceptions of the ‘problem’ and what ‘solving’ it would look like were particularly identified as risks if programmes were developed and/or implemented by those outside of a community.

*“What we quite often see is that non-indigenous people will come into the community, decide that this community needs rules about animal management, and that rule’s going to be that you must confine your dogs to the yard and if they are out of the yard they must be on a leash…. And that really changes the culture of the community… It’s not up to indigenous people to control their animals as much as we have that sense of ownership in a western world.” (DPM1).*

### 3.2 Not a silver bullet

This theme captures experiences of sterilisation programmes not leading to expected outcomes and perspectives on why this might be.

#### 3.2.1 The sterilisation treadmill

Many programmes had initially been very focused on sterilisation, with the assumption that as increasing numbers of dogs were sterilised that this would stabilise and then eventually reduce the population over time. It was only through experience of implementing a programme that it sometimes became apparent that this may not be the case. One participant summed this up as *“…we could spay and neuter until we are blue in the face, but it’s not going to change anything.” (DPM3).*

Even in areas with long term interventions and relatively high sterilisation coverage, numbers were commonly seen to plateau, after which it was difficult to make any further inroads in decreasing the total number of dogs, or increasing the percentage of sterilised dogs, despite sustained efforts: *“If you look at the graph of the population…it came down pretty steadily and then levelled off. And if you look at percentage of bitches spayed, that went up very quickly and then levelled off and it’s been really quite difficult to get it to go very much higher than sort of about 80%.” (DPM6).*

Suggested explanations for this were a continual influx of new dogs from migration, importation, increased reproductive success and/or survival of puppies and abandonment. The relative importance of these factors varied between intervention areas.

*“And we are still finding a lot of animals which really highlights to us that there’s a lot of abandonment… this is the biggest problem I think. Huge abandonment of animals and puppies” (DPM9).*

Effects of a population of inaccessible dogs, both in terms of dogs that it was not possible to catch, and owned dogs that the owners did not want sterilised, were also described.

*“[we are] certainly having more trouble catching dogs…and I think that is partly because many of the easily catchable dogs have been caught. And many of the ones that remain are those that live on waste ground that are desperately difficult to get hold of.” (DPM6).*

Increasingly the influence of other factors on dog population size and dynamics was being recognised. For example, environmental factors such as waste control affecting resource availability and therefore the number of dogs that could be sustained in an area.

*“The municipality need to have a good plan for the control of garbage because in any city you see if there is management of the garbage then it will control the dog population in the city as well.” (DPM7).*

#### 3.2.2 Change beyond numbers

Participants highlighted that concerns about FRDs extended beyond population numbers and could encompass human safety, dog welfare and wildlife conservation. This was reflected in the multiple, interconnected impacts that programmes were often trying to achieve. One participant described*“…the biggest concern is safety. All the time you have got concerns about people getting bit and dog attacks and things like that and there have been some maulings that have resulted in deaths unfortunately.” (DPM3).* Other participants emphasised that their programme goals focused on improving human health and wellbeing: *“the intention… has always been about both improving animal health and welfare but also improving community health and wellbeing… People are living in very close association with their dogs and so zoonoses and public health issues are a significant concern.” (DPM1).* Another participant highlighted conflict with wildlife as the key motivating factor for their DPM programme: *“…probably the biggest one here of concern, to us anyway, is the wildlife interaction, there’s some endangered species here and the number one issue is always reported to be dogs, attacks and diseases.” (DPM4).*

However, it was recognised that these outcomes would not necessarily materialise, even from well planned and executed sterilisation programmes and even if population reduction was achieved, as sterilised dogs can still have poor welfare, transmit disease, and cause conflict with humans and wildlife: *“I think it’s kind of looked at as the cure all, end all… and I think it’s bad because sterilised dogs still bite people, they still defecate in public areas, they still have diseases.” (DPM4)*. Furthermore, if population reduction did occur it also had the potential for adverse effects if an unmet demand for puppies led to importation of new dogs which may be less suited to the area and could potentially bring in diseases: *“If you spay/neuter every single dog in that village and then all of a sudden their old dog dies and there’s no more puppies being born in the village, they will go out and bring in new puppies. And those new puppies are often breed dogs from the markets… often bringing in you know parvo, distemper or other [diseases]” (DPM2).*

### 3.3 Looking beyond the dogs

This theme illustrates the increasing awareness of the importance of people and their attitudes and behaviour towards FRDs in achieving change.

#### 3.3.1 A means, not the end

Participants described a relatively recent shift in programme objectives, in which changes in the dog population were seen as a step on the way to achieving human behaviour change, rather than as an end in itself.

*“The biggest goal is to see a change for animals, the way that people treat animals… a behaviour change in people, that’s a long term goal to improve animal welfare…. [reducing the dog population] that’s not the ultimate goal. It’s a means, and it’s one of the ways that we are working to kind of change things.” (DPM9).*

A significant amount of the time spent describing benefits that participants had experienced from conducting sterilisation programmes were focused on changes in the human population rather than in the dog population. Sometimes this was contingent on dog population changes, e.g., visible improvements in number, health and behaviour which were then linked to a subsequent positive improvement in how FRDs were perceived.

*“…if there’s too many animals they are going to be a nuisance and of course you are not going to value those animals as much as if they are the right number that you actually want and they are healthy and they are contributing positively to your life.” (DPM1).*

#### 3.3.2 From co-existence to care

The concept of FRDs being more accepted once sterilised, and as a result, more likely to be cared for in some way and potentially less likely to be mistreated, was a recurring finding.

*“I do think that because the dogs are sterilised that has to do with why people are caring for the dogs more. I think there’s a thing that they like healthy, plump, disease free dogs, rather than skinny mangey dogs that are producing puppies all the time” (DPM12).*

It could also increase the types of behaviours conventionally associated with ownership which potentially was a sign of improved care.

*“When we first started people would form a line to register and we would ask them what’s the name of the pet? Oh, he does not have any name. Nowadays every single animal has a name.” (DPM10).*

It was considered that this created a positive feedback loop whereby the dogs were perceived to have more value and so if they were sick or injured then veterinary care would be sought. Consequently, there was then more demand for sterilisation.

*“We would typically have people going door to door throughout the community to talk with the… residents and get their permission to treat their animals… now we get more and more people coming to the surgery site themselves and proactively engaging in that service.” (DPM1).*

However, other components of the intervention were also important for facilitating and maintaining these changes in human attitudes and behaviour. For example, having access to additional veterinary services was seen to create an enabling environment, whereby there was an option for people to help sick and injured animals, and therefore less of a barrier to creating a bond with a dog.

*“I think before people just thought I’m not going to get involved because I cannot really do anything… So I’m just going to pretend it’s all not happening and not create an emotional feeling with that animal, because if they are sick or injured, I cannot do anything about it anyway… Whereas I think now they do feel empowered that okay, I am going to love this animal because if something does happen to them, there are options available to me as to what I can do to help them…” (DPM12).*

These changes in how animals are viewed and treated was seen as a key outcome which was perceived to lead to more long term and sustainable changes in the dog population than the direct effects of the sterilisation.

*“We are actually starting to see now… some of the dogs that we met in the beginning of our organisation they were adult dogs already and so they are passing away now… and people are getting new puppies and they are instantly reaching out right away, I really want to get my dog fixed.” (DPM3).*

Paradoxically, the effects of an increase in ‘positive’ animal welfare behaviours, such as feeding unowned dogs, could potentially have unwanted effects on population dynamics.

*“I think the more people you have got feeding the dogs, the higher their fertility becomes. I do think more dogs are having two litters of puppies a year, rather than one…. I do not know how much of that is in response to trying to fill any biological niche, or how much is in response to being healthier and having more food… but… it’s like… stop breeding! We’re not making you healthier so you can have more puppies.” (DPM12).*

However, it is important to note that behaviour change was not a linear nor a uniform process, and opinions on FRDs could still be extremely varied. There were often conflicting opinions (as described in ‘Seeing only part of the problem: *different perspectives*’) centred around the question of the ‘place’ of dogs.

*“In some communities they object violently to us taking the dogs away. And in some, there are considerable objections to us taking them back.” (DPM6).*

### 3.4 Sterilisation within a wider strategy

This theme describes key facilitators related to how a programme was implemented that were considered important for success.

#### 3.4.1 Co-creating change

Central to this theme was the need for a long term outlook that would lead to sustainable changes. There was recognition that this required a more holistic approach rather than just dog-focused interventions: *“[The aim is to] hopefully create something that’s long term and not just these continual band aids as just going in and removing dogs or just spay and neuter, it does not work…” (DPM3).* It also required engaging the community in a meaningful way, which often had not been considered a priority when programmes began.

*“It was coming in and sterilising… Our old dog catchers I tell them all the time can you stop being like a UFO! They were used to the old way, which was more about just getting the job done… it was overwhelming, you come and you have got plenty of dogs around every corner, and the motivation is like let us just get this done…” (DPM9).*

Participants also recognised that it was important to manage stakeholder expectations regarding impacts and time frames, as interventions could be counterproductive if there had been an unrealistic expectation of what was to be achieved, and this had not then happened. One participant suggested that for this reason, interventions should only be implemented if there was the capacity for a large scale programme: *“…it is better to not start if you are not well prepared… otherwise the people and municipality or the other authorities will get disappointed… they [will] say you started dog population management and you told us to stop killing dogs and… your programme is not getting anywhere for 3,4,5 years, and still the same.” (DPM7).*

However, other participants described the incremental changes that could be seen from being a constant (or regular) presence in an area, which helped to build trust and could then lead to increased engagement with, and support for, an intervention*: “It’s been mostly word of mouth… The community members are saying yeah they helped my dog 2 years ago they are great, we are so happy they are here, and so experientially we have seen that grow over the years where initially a community may have been reluctant to have their animals have surgery… to the point where they are requesting more spay/neuter services.” (DPM8).*

However, this could be difficult and take time to rebuild if the local community had been disenfranchised from prior experiences, leading to a reluctance to engage in subsequent initiatives: *“I think the biggest reason for rejection is because they are frightened [the dogs] will not come back. Because their experience with the government agencies that collect dogs, local government, are that on many occasions these dogs are picked up and taken somewhere else, dumped in the jungle or whatever…” (DPM6)*. Building effective relationships and working together in collaboration with other stakeholders was seen as a key component for facilitating sustainable changes.

*“The main thing is about collaboration and involving the community, working closely with people, because otherwise it’s like you were not there… what’s the point of investing all this time and money and energy into something that when you are not around a couple of years later it goes back to being back as it was before.” (DPM9).*

This included trying to ensure that local government and other stakeholders were at the very least aware of the problems that existed, and what was being done: *“[when we get a rescue call] we tell them to call the municipality first because if the municipality does not understand that the problem is still there, if [we] try to solve all the problems, then they might be thinking that there is no problem at all…” (DPM5)*. The ideal situation was that programmes were integrated into local governance structures. However this required aligned perceptions and goals, particularly from a government and policy perspective. Where this was not the case, there could be concurrent or subsequent use of strategies that were in conflict, e.g., removing or culling dogs. This could impair the effectiveness of a sterilisation programme if sterilised and vaccinated dogs are removed leaving an ecological niche which could be filled by unsterilised and unvaccinated dogs.

*“It’s an idea that came from the government…. they do not want to see any stray dogs… This idea that you can kind of put them all in shelters is just ridiculous… So they do sometimes round up random dogs… And it’s frustrating because usually they are all sterilised… they have been there for a while and they are keeping rabies out and they are safe dogs and they should be there but they are not.” (DPM9).*

This links back to ‘Seeing only part of the problem: *different perspectives*’ and the contrasting viewpoints regarding FRDs and therefore what the ideal outcomes of an intervention might be, and was one reason mentioned as to why collaboration could be difficult. In one country, legal restrictions prohibited the release of dogs so programmes were not permitted to sterilise dogs and return them to their territory. There was a sense that whatever successes were achieved locally that bigger changes were needed for more sustained and wider progress.

*“It’s just not having that collaboration, not having people on the same page… the government specifically, they are in a role that could potentially turn this around quickly… If you look through time at some of the most important things that we have changed… like smoking, and drinking and driving… that changed in a fairly short period of time… they did it through public outreach campaigns and… through the law” (DPM4).*

#### 3.4.2 Part of developing veterinary capacity

Developing veterinary capacity in an area was seen as an important long-term goal that was necessary to sustain change. As already discussed in section ‘Looking beyond the dogs: *from co-existence to care*’, additional veterinary services could be important for facilitating and maintaining changes both in dog health and welfare and in human-dog relationships.

*“We’re also vaccinating the dogs for parvo and distemper because we would go in and we would sterilise dogs and treat their skin conditions and their worms and then they are all dying of parvo or distemper.” (DPM2).*

Working together with stakeholders from the veterinary profession was seen as beneficial in improving veterinary capacity and standards. Training of lay staff, e.g., as dog catchers, vaccinators or animal health assistants also helped capacity building. This could be either as direct employees of the programme or through training of municipality staff.

*“Previously these municipality staff, everybody was calling them the dog killers and now they say proudly we are animal welfare officers… Some of them were scared of dogs… they lost their colleagues to rabid dog bites… And now they are fully vaccinated and they know the behaviour of dogs…” (DPM7).*

Private veterinarians were seen to benefit from the presence of sterilisation programmes through an increase in overall demand for veterinary services. Vets who were trained or worked within interventions sometimes went on to work in these clinics or in government veterinary services. This was seen to be a positive step in driving up the standards of veterinary care on a wider scale than just in the immediate intervention area.

## 4 Discussion

This study provides novel insights into the use of sterilisation in DPM interventions as reported by those working in the field. We show that the management of FRDs should be recognised as a complex problem (Theme 1) which explains some of the limitations to what can be achieved through sterilisation alone (Theme 2). The importance of human factors (Theme 3) in both contributing to the problem and needing to be part of the solution was highlighted. This included changes in the behaviour of those implementing such programmes in thinking beyond sterilisation numbers and instead planning long-term approaches which emphasise engagement, collaboration and building local capacity (Theme 4).

The experiences and perspectives that the participants described aligned with many of the features of a ‘wicked problem’ (37). In contrast to a tame problem, which has clear boundaries and a clear solution, a ‘wicked problem’ is complex, difficult to define, and does not have a clear solution. A number of these descriptors are especially relevant in relation to management of FRDs and will be summarised in a separate paper. Reframing FRD management as a ‘wicked problem’ emphasises that there are no quick-fix solutions (37). This would help to avoid over-simplification of the problem, which can lead to a mismatch between programme activities and expectations (38). The focus on sterilisation within DPM could be seen as an example of the tendency to focus on a manageable part, or the symptom of a ‘wicked problem’, rather than addressing the root causes (37, 39).

Each programme was unique in terms of their scope, priorities, capacity, resources, approaches and strategies used. This reflected both organisational differences and context specific factors such as the geographical area, human-dog relationship, legal and regulatory frameworks, dog population demographics and availability/access of other animal health services. Understanding the ways in which context, implementation and setting interact are important (40). Organisations appeared to adjust the practice of sterilisation according to their own specific contexts and these variations appeared to trigger different pathways to change. For example, whether dogs were predominantly unowned or owned in area affected certain logistical considerations, e.g., how the dogs were located or brought in for surgery, but also in priorities for outcomes in terms of population vs. individual level effects on the dogs. This suggests that sterilisation may work differently in different contexts and, as a result, its implementation cannot be assumed to have the same effects in different settings (41). However, certain factors such as the importance of building trust, fostering true community engagement and wider institutional support were seen as facilitators across the diverse contexts represented in this study. These findings reflect those recently found in unowned cat population management interventions (42) which face many of the same challenges, and which call for integrated governance frameworks that align legal authority with implementation capacity and public support in order to produce sustainable outcomes. Legislation and enforcement were not widely discussed during the interviews, however they are one of the key foundations for effective DPM (10) and can help create the conditions in which community engagement and behaviour change can occur.

One key finding from this study was that many of the challenges associated with managing FRD populations can stem from an incomplete or narrow understanding of the nature of the problem. As a result, sterilisation programmes are often implemented in a reactive way without collection of baseline data, clear objectives or sufficient consideration of the wider system of influencing factors such as migration, abandonment and resource availability. This is supported in other studies (43) alongside recommendations that effective planning requires some method of enumeration and characterisation of the FRD population (10), understanding the processes that establish and sustain it (13), and clarity about the intended outcomes (44). Managing expectations can be complicated by divergent perspectives on prioritisation of concerns, e.g., animal welfare, public health risks, or impacts on wildlife, and therefore consequently, on what the desired long-term outcomes of intervention should be. One approach that could be considered, and that has been utilised for other complex interventions with similar challenges, such as wildlife conservation, is adaptive management. This involves an iterative cycle of goal-setting, design and planning, implementation, monitoring, evaluation, and adaptation, and developed from a recognition of the limitations of traditional scientific approaches in understanding complex social-ecological systems (45).

The ethical considerations of balancing animal welfare, the human dimensions and ecological concerns must also be considered in intervention strategies. This was highlighted in participant accounts, in terms of diverging stakeholder perceptions of ‘success’, and particularly where interventions had been instigated or were managed by those outside of a community. As shown by (46), differing problem framings reflect distinct but also multi-dimensional understandings, often around the ‘place’ of dogs in society, and can mean that intervention priorities may not reflect community concerns. The data emphasised that programme success was closely linked to how interventions were developed and implemented, and the need for locally led, context specific, participatory approaches. The practice of imposing external ideas and preconceived goals regarding FRD control, should be avoided and instead, bottom up approaches that complement and support local capacities should be emphasised (47). This shift requires acknowledging that “well-intended interventions framed by Western subjectivities and objectives can disempower local actors and re-entrench colonial power relations” (47).

Participants reported many benefits that they attributed to their sterilisation programme, such as effects on dog health, welfare and in human perceptions and behaviour towards dogs. However, our data suggest initial beliefs regarding expected effects on the dog population were often challenged. Participants described population control goals as difficult to reach and/or maintain, even in areas that had implemented sterilisation over a sustained period, with high levels of coverage. This is supported in other studies where the influences of factors such as abandonment and/or migration (20, 21, 48, 49) have been found to mitigate the population impacts of sterilisation efforts. One study in India found substantial decreases in the number of sterilised dogs each year suggesting high mortality and migration of sterilised dogs (50) indicating that sustained efforts are needed in order for effects to be maintained. This aligns with the need for long term monitoring and evaluation as an intrinsic part of programmes, which was highlighted in this study, and is a crucial part of adaptive management, as discussed above.

Much of the literature on sterilisation in DPM focuses on quantitative changes in the dog population through observational, experimental or modelling studies (11), and has been focused on demonstrating if sterilisation ‘works’ or ‘does not work’ in terms of changes in dog population numbers. However, the findings suggest a conceptual shift in DPM, with participants increasingly viewing changes in the dog population not as the ultimate goal but as a means to support improved dog-human co-existence. For example, in some settings sterilisation programmes were seen as beneficial in creating an enabling environment for animal welfare and fostering changes in human attitudes and behaviour that could lead to increased welfare going forwards. This arguably was more important for sustainable changes than the direct effects on the dog population. Furthermore, research on attitudes and ownership practices illustrates that human beliefs, cultural context and behaviours influence how communities engage with population management strategies and can both facilitate or constrain intervention outcomes (51). Therefore, mixed-methods, systems-thinking approaches, such as those that have been developed to evaluate One Health initiatives (52) could provide a more complete understanding of interventions, their context and their interconnections. In addition, the use of realist evaluation frameworks, that aim to identify ‘what works, in which circumstances and for whom?’, rather than simply answering ‘does it work?’ could be considered (53). This approach could improve the translation of evaluation findings to other programmes and settings, through the identification of underlying mechanisms that have driven any changes.

The ecological aspects of free-roaming dog population management were touched on by some of the participants, e.g., in terms of resource availability and effects on wildlife, but they were not discussed to the same extent as the human dimensions. There could be several potential reasons for this such as geographical locations meaning that ecological implications were not considered to be a key issue concerning the FRD populations in this study, the organisations that participated may have prioritised other aspects such as dog welfare or zoonotic disease risks to humans, or it may be that these aspects are less well understood or integrated into many programmes. Only one participant stated that their main reason for instigating DPM was because of the impact of FRDs on wildlife. However, there is growing evidence of the negative ecological impacts of FRDs. This can include predation, disturbance, disease transmission and hybridisation (7, 9). Sustainable management will require attention to ecological factors, both in terms of shaping dog populations as well as the wider consequences, such as wildlife conservation and other environmental concerns.

The current focus on sterilisation could potentially hinder progress in DPM if it means that additional or alternative strategies are not considered, or if programmes are implemented without the considerations identified, i.e., local ownership and a comprehensive understanding of the issues. As for other ‘wicked problems’ in complex socio-ecological systems, challenging existing strategies and beliefs is needed (54) and might include expanding the boundaries of the problem so that a wider range of options for action may develop (55). These findings have important implications for those working in DPM, particularly those for whom sterilisation is a large component of their programme. This is in accordance with WOAH’s standards which now define DPM as a holistic approach that aims to improve the welfare of dogs, reduce problems they may present and create harmonious co-existence with people and their environment (3).

### 4.1 Limitations

As this qualitative research involved a small number of DPM practitioners from a limited number of programmes and countries worldwide, these data cannot be taken to be representative of all DPM practitioners or programmes. Although the study includes programmes from multiple regions, the absence of participants from Africa and the Middle East limits the geographical breadth of perspectives represented. These regions have distinct socio-ecological, cultural, and governance contexts for free-roaming dog management, and their exclusion means the findings may not fully capture the diversity in global DPM. Furthermore, even within countries and included in the study, contextual conditions, such as organisational structures, resource availability, political environments, and community-animal relationships, vary substantially between programmes. The use of purposive sampling enabled the recruitment of experienced practitioners working in a wide range of contexts, who could provide insights and in-depth understanding, however limits generalisation of the findings. Participant selection may have introduced bias, as individuals who were approached and who agreed to participate may possess certain characteristics or experiences that do not represent the broader population of DPM practitioners. For example they were all English speaking (as a first or additional language), most had already participated in our previous research study and so were already interested in, and reflecting on the impact of their programme.

Interviews relied on practitioners’ recall of their programme including data collected and other perceived changes in intervention areas, which may have been incomplete or biased, and may have been subject to social desirability bias, particularly in terms of perceived successes or failures. Within the dyadic and triadic interviews there was a potential for hierarchical influence and reduced openness. However, the questions were designed to be broad and descriptive and did not touch on sensitive or personal information. In addition, it was discussed at the start of the interview that all viewpoints are valued and that there were no ‘right’ or ‘wrong’ answers to try to encourage open contribution (56).

Despite these limitations, this report identified many commonalities across multiple diverse settings. However these were only from the perspective of practitioners working within DPM programmes. Other key stakeholders in FRD management, such as local communities, policymakers and public health officials are likely to have different perspectives to the practitioners interviewed in this study. The methods applied could form the basis for future work with these other groups, which would enable a more comprehensive understanding of the topic.

## 5 Conclusion

Management of FRDs may be usefully understood as a ‘wicked problem’ which is complex and multifactorial and does not have a simple solution. Therefore, there are limitations to what sterilisation can achieve on its own and additional strategies addressing other influential factors, particularly the human dimension, are required. The findings indicate a transition away from dog population focused outcomes towards strategies that foster human-dog coexistence. Programmes need to ensure they have a comprehensive understanding of the problem including community concerns and priorities, define their own goals and tailor strategies to their specific context. These insights can be used to help develop sustainable interventions that address these complexities and are appropriate to both the dog populations and the communities within which they live.

## Data Availability

The datasets presented in this article are not readily available because they consist of interview transcripts that contain potentially identifiable information. Requests to access the datasets should be directed to Marnie L. Brennan, marnie.brennan@nottingham.ac.uk.
