# Supplementary material for: “It’s just one piece of the puzzle…”: practitioners’ perspectives on the use of sterilisation in the management of free-roaming dog populations
Source: Front Vet Sci. 2026 Aug 18;13:1898448. doi: 10.3389/fvets.2026.1898448 (PMC13529531; doi:10.3389/fvets.2026.1898448)
Supplement: Supplementary file 1 [file Supplementary_File_1.docx]

# Supplementary material

Interview guide

1. Questions about the organisation:

- Why it was started/main concerns with FRDs in area?
- What activities are conducted?
- Have you changed the way you work in any ways since the organisation started?
- What are your reasons for using sterilisation specifically?
- What are your main long term goals?

2. Overview of the sterilisation component

- How does the sterilisation programme work (from accessing dogs to returning/releasing)?
- What would you consider to be the main challenges/constraints to running the programme?
- What do you think helps the programme work well?
- Is there anything else that affects the programme?
- Is there anything that means that the programme cannot be run in the way you would like?

3. Outcomes and impacts

- What would you say have been the main successes so far?
- Have there been any unexpected changes?
- Are there any negative impacts you can think of?
- What other factors do you think may also have had an effect on these changes?
- Have there been any planned or expected changes that have not happened?
- What do you think has hindered progress to expected changes?
- In your opinion, what are the main barriers to your programme achieving its goals?
- In your opinion, what are the main problems that still exist concerning FRDs?
- What changes do you think would have the biggest impact on reducing problems associated with FRDs?

4. Monitoring and evaluation:

- How do you monitor progress?
- What data is collected? (if any)
- What are the challenges to collecting data?
- What is done with data collected?
- Is there anything else that you would like to measure?
- Can you think of any examples of how you have seen the programme working in action that may not be captured by data collection?

5. Is there anything else that we haven’t discussed that you think is important to mention?
